# Regulatory considerations in the design, development and quality of monoclonal antibodies and related products for the diagnosis and treatment of cancer

**DOI:** 10.3389/fonc.2024.1379738

**Published:** 2024-04-30

**Authors:** Marjorie A. Shapiro

**Affiliations:** Office of Pharmaceutical Quality, Center for Drug Evaluation and Research, US Food and Drug Administration, Silver Spring, MD, United States

**Keywords:** monoclonal antibody, bispecific antibody, antibody drug conjugate, Fc-engineering, immunogenicity, isotypes, radioimmunoconjugate, antibody-fusion protein

## Abstract

Over 160 therapeutic and *in vivo* diagnostic monoclonal antibodies have been approved by the US FDA since the first monoclonal antibody, muromonab, was approved in 1986. Approximately 42% of these approvals were for the treatment or *in vivo* diagnosis of oncology indications, although some products are no longer marketed. This review will look at the history of monoclonal antibody development and approvals, discuss current antibody-based modalities, regulatory considerations for engineering approaches, critical quality attributes for different modalities, immunogenicity of mAbs across oncology products, and the future directions for development of therapeutic and diagnostic monoclonal antibody-based products.

## 1 Introduction – history of monoclonal antibody approvals and antibody engineering

The first therapeutic monoclonal antibody (mAb), muromonab, was licensed in 1986 for the treatment of acute allograft rejection in renal transplant patients, but it wasn’t until the late 1990s that the potential of therapeutic mAbs began to be realized. Following the licensure of muromonab, 12 mAbs were approved in the 1990s; 7 therapeutic mAbs (2 for oncology) and 5 imaging mAbs (4 for imaging tumors to determine the location and extent of the tumor).

The first therapeutic mAb for an oncology indication was rituximab, approved in 1997 for the treatment of patients with relapsed or refractory low-grade or follicular, B-cell non-Hodgkin’s lymphoma. Next was the approval of trastuzumab in 1998 for the treatment of patients with metastatic breast cancer whose tumors overexpress the HER2 protein and who have received one or more chemotherapy regimens for their metastatic disease. Since their original approvals, both products have added additional indications, including autoimmune indications for rituximab, and both have had a major impact in improving outcomes in the oncology indications.

The early failures of most mAbs were attributed to insufficient characterization of the specificity and function or intended mechanism of action (MOA) of the mAb, poor preclinical development, inadequately designed clinical trials and poor target selection (1, 2). In addition, the first mAbs used clinically were derived from murine hybridomas, which have a short half-life in humans, are inefficient at eliciting effector functions, and induced human anti-mouse antibodies (HAMA) (3, 4).

The lessons from these early failures led to the genetic engineering of chimeric mAbs, murine or other non-human variable regions (V region) expressed with human constant regions. This was quickly followed by the development of humanized mAbs, which contain murine or other non-human complementarity determining regions (CDRs) grafted onto human framework regions of V region genes expressed with human constant regions. In direct contrast to their murine counterparts, chimeric and humanized mAbs are predicted to be less immunogenic (5), exhibit longer half-lives, and efficiently promote effector functions in humans (3, 6, 7).

Subsequently, the development of mAbs containing entirely human-derived sequences was facilitated by two approaches: the engineering of transgenic mice to express germline human heavy and light chain genes (8–10); and the development of display technologies to express human antibody genes derived from human donors or constructed synthetically based on human germline gene sequences and the frequency of their use (11, 12). Human mAbs share the advantages of chimeric and humanized mAbs regarding Fc-mediated effector functions and half-life and are predicted to be less immunogenic than chimeric and humanized mAbs. Today, many anti-viral mAbs and a few mAbs directed against human targets are isolated directly from human B cells (13, 14). With B cell repertoire profiling in tumor microenvironments (TME) and in some cases expressing antibodies that contribute anti-tumor activity, isolation of B cells from cancer patients may become a source of novel mAbs for oncology indications (15–17).

Most approved mAbs use an IgG isotype, although several are antibody V region fragments (Fab, F(ab’)_2_, scFv, single domain abs). V region engineering progressed beyond reducing immunogenicity to include the optimization of affinity, developability and manufacturability (7, 18, 19), and to extend half-life (20). Studies early in product development help identify potential amino acid sequences that could result in unfavorable quality attributes, such as aggregation-prone motifs, immunogenic motifs and amino acids prone to post-translational modifications, including V-region glycosylation (7).

IgG1 is used if the MOA includes Fc-mediated effector functions, whereas IgG2 and IgG4 are used when effector functions are not desired. One of the earliest types of Fc engineering is the S228P mutation in the IgG4 hinge region to prevent Fab arm exchange with endogenous IgG4. Gemtuzumab ozogamicin, which was developed in the 1990s and initially approved in 2000, was the first approved IgG4 mAb and incorporated this mutation (21).

Amino acid residues in IgG1 that bind to complement, FcγR or the neonatal Fc receptor, FcRn, have been identified and mutated in mAbs to achieve the desired outcome. Some mutations enhance binding to FcγRIIIA for improved antibody-dependent cellular cytotoxicity (ADCC) and/or to FcγRIIA to improve antibody-dependent cellular phagocytosis (ADCP) activities, while other mutations that reduce binding to FcγRs are used when effector functions are not wanted (22–25). Cell line engineering is also used to produce afucosylated glycan structures to enhance ADCC (26).

Some mutations may increase binding to all variants of FcγRII and FcγRIII, while others may increase binding to FcγRIII but decrease binding to FcγRII, particularly the inhibitory receptor, FcγRIIB (24). Margetuximab, an anti-HER 2 mAb approved in 2022, was Fc-engineered to enhance binding to FcγRIIIA and reduce binding to the inhibitory receptor, FcγRIIB (27). It did not have an overall survival advantage over trastuzumab treated HER2-positive advanced breast patients but did show an advantage in the subset of patients who were homozygous for the lower affinity FcγRIIIA-158F variant (27, 28). Additional pre-clinical and clinical studies will be needed to understand the lessons to be learned from the Phase III clinical trial.

Increasing binding to FcγRIIB has been shown to improve the performance of agonist immunostimulatory mAbs, likely due to cross-linking agonist mAb bound to its target on the cell surface (29, 30). Many reviews provide a more in-depth discussion of isotype selection, Fc engineering approaches for FcγR, FcRn, or complement binding and the mechanisms by which these modifications achieve the desired outcome to promote efficacy and safety of the mAb (22–25, 31, 32).

**Figure 1:**
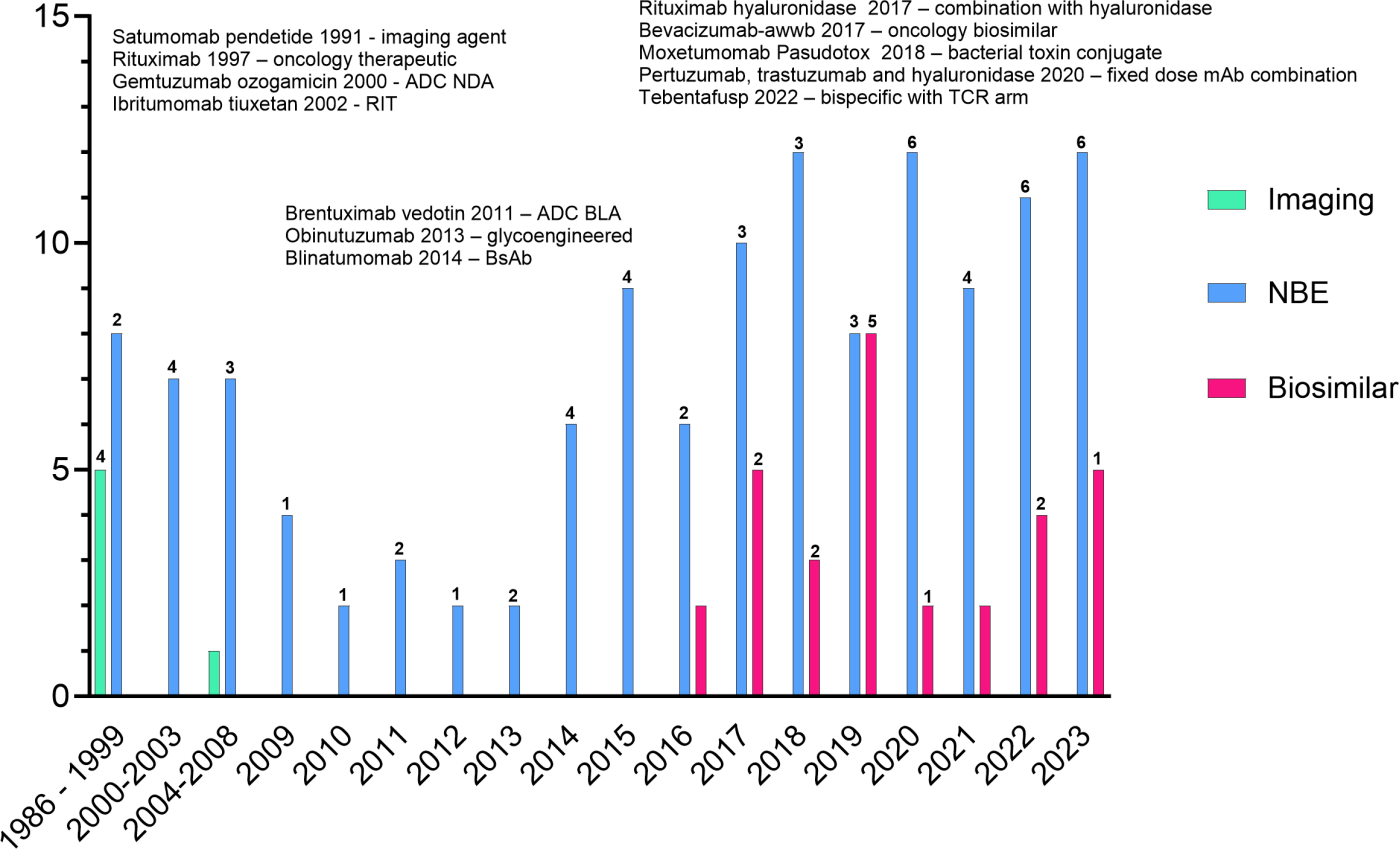
All FDA approved monoclonal antibodies. Products listed indicate firsts for oncology products. Numbers above the bars indicate the number of oncology approvals in that year. The graph does not include the approvals of rituximab hyaluronidase or the combination of pertuzumab, trastuzumab and hyaluronidase as the individual components are not novel.

Many approved products are no longer marketed; however, by the end of 2023, over 160 novel and biosimilar mAbs were approved by the FDA (**Figure 1**). Approximately 42% of the approvals are for oncology indications: 4 imaging agents, 12 antibody-drug conjugates (ADCs, including one bacterial toxin conjugate), 9 bispecific antibodies (BsAbs), one co-formulated mAb combination, and 13 biosimilar mAbs. Several of the approved products incorporate Fc-engineering or are expressed in glyco-engineered cell lines. While most mAbs for oncology indications are delivered by intravenous infusion, several products have been reformulated with recombinant human hyaluronidase for subcutaneous injection. These products allow a shortened administration time compared to the time needed for intravenous infusion (33).

## 2 Quality target product profile and critical quality attributes

Since a rational design can be applied to antibody-based products (34, 35), developers should formulate a quality target product profile (QTPP) early in development that considers the intended clinical use, dosage form and strength, route of administration, delivery systems, and critical quality attributes (CQAs) that impact sterility, purity, stability, safety, efficacy, and pharmacokinetics. A CQA is defined as “a physical, chemical, biological, or microbiological property or characteristic that should be within an appropriate limit, range, or distribution to ensure the desired product quality.” (36).

It is expected that the MOA of mAb based products will be characterized to the extent possible. All mAbs, including ADCs, BsAbs and mAb-fusion proteins must bind their target. Characterization of a mAb should determine the affinity for the target, including on- and off-rates, and binding of the homolog from other species for non-clinical assessments. Depending on the antigen, antigen density and indication, the affinity for antigen can influence effector function (37) and affect localization and tumor penetration (38, 39). In some cases, reducing affinity of mAbs may result in fewer or less severe adverse events, such as the anti-CD3 arm in BsAbs (40, 41), or enhance activity of immunomodulatory products (42).

All mAbs, including ADCs, BsAbs and mAb-fusion proteins, should be assessed for their ability to carry out Fc-mediated effector functions (43). This can be done once to demonstrate the effectiveness of the engineering for mAbs with low to no effector function, but for mAbs intended to have effector function, assays to assess this activity should be performed routinely.

One of the biggest gaps in knowledge for mAbs with Fc-mediated effector functions is demonstrating a specific effector function *in vitro* and understanding what the predominant mechanism may be *in vivo* for each indication. In addition, some adverse events, such as first infusion reactions, may be related to engagement of FcγR on neutrophils (44), however infusion reactions can be managed.

In patients, the predominant Fc-mediated mechanism will depend on the effector cells at the site of the tumor, the FcγRs expressed on those effector cells, or if tumor cells express complement inhibitory proteins. It is also dependent on the epitope of the target antigen: epitopes closer to the membrane favor ADCC activity while epitopes further away favor antibody-dependent cellular phagocytosis (ADCP) activity (45, 46). Antigen density (37, 47), as well as the angle of binding the antigen may influence MOAs, which has been shown for rituximab and obinutuzumab (48, 49). Optimization of the Fc-region for a specific oncology indication should consider the target (tumor antigen versus immunoinhibitory or immunostimulatory receptors) and the types of effector cells and the FcγRs they express in the TME (31, 32).

Despite this uncertainty, it is important to distinguish potential *in vivo* MOAs from assays used for quality control (QC) of a product. The MOA of a mAb and features that may impact binding to its target, such as glycosylation (50) and other post-translational modifications (PTMs) in the CDRs (51–53) and constant regions (54), are considered CQAs. The absence of an Fc-glycan reduces complement dependent cellular cytotoxicity (CDC) activity and abrogates FcγR-mediated activity (55), but more detailed knowledge of the impact of specific glycan structures on Fc-mediated effector functions, specifically afucosylated glycans, became known after the approval of several mAbs, including rituximab, trastuzumab, alemtuzumab and cetuximab (56–59). It is well established that afucosylated glycan structures have enhanced ADCC activity because afucosylated IgG1 binds better to FcγRIII than fucosylated IgG1 (58) and that galactosylation, high mannose and sialylated forms also contribute, but not to the same extent as afucosylated species (60–67). However, there is likely still more to learn about the effect of specific glycoforms on ADCC and other Fc-mediated effector functions.

If a mAb has multiple Fc-mediated effector functions, or if the predominant mechanism is unknown or may differ per indication, it may not be feasible to assess all mechanisms for product release and stability testing. The purpose of QC testing is to ensure the consistency of each lot of a product. Therefore, a potency assay or assays for release and stability that reflect a relevant MOA, if not the predominant mechanism, should be developed and all attributes that are thought to affect the MOA should be adequately controlled in this assay or by other methods. Historically, complement dependent cellular cytotoxicity (CDC) assays were used since cell lines were not available for ADCC assays and using effector cells isolated from donors is challenging for QC purposes. CD16-expressing cell lines or surrogate reporter-gene ADCC assays became available in the last 10-15 years, so now most mAbs with effector function include an ADCC assay for release. ADCP assays are still challenging but improving. Many are in development, including reporter gene surrogate assays (68–70).

Using an FcγRIIa surrogate assay, Kuhns et al. (70) also showed that terminal galactosylated, afucosylated and high mannose glycoforms have a different or no impact on ADCP activity, depending on the IgG1 mAb and target antigen. In contrast, the effect of different glycoforms on ADCC activity appears to apply across IgG1 mAbs, regardless of target. More work is needed to fully understand the relationship between Fc-glycan structures and ADCP. Furthermore, since there appears to be opposite effects of some glycoforms on ADCC and ADCP activity, for each mAb it is important to understand which mechanism is likely the predominant activity for a given indication and to optimize the glycoforms for each mAb that may favor one activity over the other or optimize the glycoforms to adequately promote both activities.

From a regulatory perspective, based on current knowledge that ADCC activity varies with levels of fucose on the N-glycan structure, ADCC assays are considered the most sensitive to detect differences among lots. Methods that assess binding to FcγR, glycan structures and other relevant PTMs can also be used to control CQAs relevant for potency. As more knowledge is gained about the relationship between glycan structures and other effector functions, regulatory expectations may change depending on the predominant predicted MOA for a mAb.

## 3 Different mAb-based formats

IgG (IgG1 >> IgG4 > IgG2) is the main antibody isotype of approved and clinical stage products. However, there are different isotypes, different types of V region only constructs and various formats of bi- and multi-specific antibodies that lack an Fc region. Engineering IgG and V region fragment-based products beyond the standard mAb format includes more complex structures, such as BsAbs, ADCs, imaging agents, and bifunctional fusion proteins (71).

### 3.1 Bi- and multi-specific antibodies

**Figure 2:**
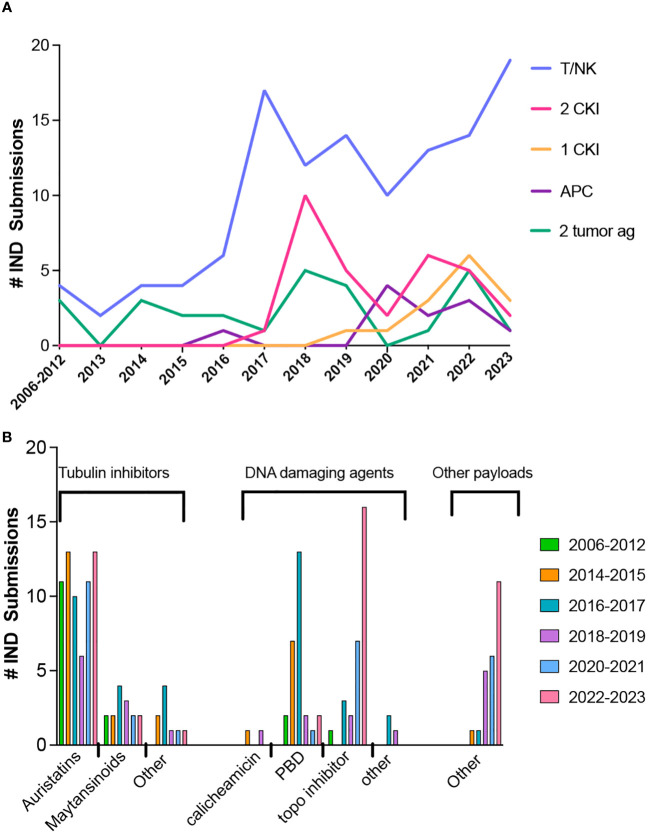
Trends in bispecific antibodies and antibody-drug conjugate IND submissions for oncology indications. **(A)** Bispecific antibody IND submissions between 2006 through October 2023. **(B)** Trends in ADC payloads from 2012 through October 2023.

For BsAbs and multi-specific antibodies (for brevity, BsAb in the remaining discussion includes multi-specific constructs), the number of novel constructs continues to grow, including those with and without an Fc region and those with either symmetric or asymmetric valencies, as described in several excellent reviews (72–77). Full length constructs are engineered to promote heterodimerization over homodimerization of heavy chains and pair the correct light chains and heavy chains (72).The majority of BsAbs in clinical development are for oncology indications and most of those products are designed to engage T cells (**Figure 2A**), while targeting a tumor antigen. More recent designs incorporate an arm designed to engage NK cells (78, 79) or antigen presenting cells instead of T cells (80). Since 2017, many products have one or two arms that target a checkpoint inhibitor or immunostimulatory molecule, with the remaining constructs targeting two tumor antigens. Among the 11 approved BsAbs, 9 were approved for oncology indications; 8 are T cell engagers (TCEs) and one targets two tumor antigens. One of the approved TCEs, tebentafusp-tebn, is a bispecific molecule containing a T cell receptor arm specific for a peptide-HLA complex rather than targeting a tumor antigen with a second antibody arm (81).

Due to the numerous platforms for BsAbs, each design has its own set of advantages and disadvantages and the CQAs will be specific for the format (82, 83). Fragment based BsAbs have a short half-life but due to their smaller size and typical lack of glycosylation, are easier to manufacture, characterize, may distribute more rapidly and uniformly to the tissues and have better tumor penetration. A disadvantage for products requiring continuous infusion due to the short half-life is sterility assurance. Full length constructs or those with additional binding domains appended to an antibody structure have pharmacokinetic (PK) profiles like mAbs and may have effector function, if desired. Disadvantages of these more complex constructs include additional characterization studies to elucidate the more complex structure, a more complex control strategy and less efficient tissue distribution.

The size and design of the BsAb affects PK parameters, which in turn impacts efficacy and safety. Constructs based on single domains may include an anti-human serum albumin (HSA) after or HSA itself to prolong half-life. The CQAs that impact the binding of anti-HSA and HSA domains should be well controlled since they would be related to the PK of the product.

Challenges for TCEs include toxicity such as cytokine release syndrome (CRS), on target/off tumor effects, especially for solid tumors, and engaging all CD3+ T cell subsets, which includes regulatory T cells and other subsets that aren’t cytotoxic for the tumor cells (84, 85). The design of a TCE BsAb can overcome specific challenges, such as mitigating cytokine release, improving biodistribution and PK parameters, and *in vivo* activity by reducing the affinity of the CD3 arm and optimizing the affinity and valency of the tumor specific arm. One way to reduce CRS is to initially use a step-up dosing strategy, which has been successful for anti-CD20 x CD3 BsAbs (86). However, a moderate or low affinity anti-CD3 arm, compared with the higher anti-CD3 affinities of the first generation TCEs, or a combination of a lower affinity anti-CD3 arm with a high affinity anti-tumor antigen arm can reduce CRS and improve efficacy (40, 41, 87). The valency and affinity of the TA arm and size of the antigen, specific epitope, distance from the membrane and antigen density (88) also affect the benefit to risk ratio of the BsAb and can be designed into the construct. However, other than perhaps the benefit of a lower affinity anti-CD3 arm leading to lower CRS, these other parameters should be studied specifically for each BsAb to determine the optimal design for the intended indication.

Newer TCE designs address on target/off tumor toxicity using a prodrug format where the TCE only becomes active in the TME. One approach designed an anti-EGFR x CD3 BsAb with both binding domains masked with a peptide linked to a protease substrate that is cleaved in the TME (89). An anti-EpCAM x CD3 BsAb is designed to address on-target off-tumor of previous anti-EpCAM products by having reduced affinity for both targets at the pH in healthy tissue and higher affinity in the more acidic pH of the TME (90). Both prodrug TCEs show improved safety in animal models compared to their non-pro-drug counterparts.

### 3.2 Antibody-drug conjugates

The FDA has approved 12 ADCs as monotherapies or in combination with standard of care therapy. For ADCs, the challenge is to find the right target for a given indication and pair the mAb with the right payload and linker to mitigate toxicity while maximizing activity. The next challenge is to find the right combination of an ADC with more targeted therapies, either small molecule drugs or other biological products, that can improve efficacy while maintaining manageable and acceptable levels of toxicity (91). CQA’s specific for ADCs include the drug to antibody ratio (DAR) and may include the drug loading distribution (DLD) depending on the conjugation strategy, free mAb and free drug. Additional CQAs will be specific for the mAb and drug-linker combination.

The payloads of the approved ADCs include 5 with DNA damaging payloads (2 calicheamicin, 1 pyrrolobenzodiazepine dimer (PBD) and 2 topoisomerase inhibitors), 6 microtubule inhibitors (4 auristatins and 2 maytansinoids) and 1 bacterial-toxin-mAb conjugate. The most common payloads in clinical trials to date are microtubule inhibitors and DNA damaging agents, but other types of payloads are in development. Trends in payloads show a steady use of auristatins, a decrease in PBD, and an increase in the use of topoisomerase inhibitors and other types of payloads (**Figure 2B**).

New payload categories include, but are not limited to, immunostimulatory molecules (92–95), which are designed to activate myeloid cells in the TME, and oligonucleotides (ON). Both mAbs and ON are successful categories of therapeutics on their own, but antibody-oligonucleotide conjugates (AOCs) combine the specificity of the mAb to its target and the specificity of the ON to down-regulate gene and subsequently, protein expression (96–98). However, the challenges for AOCs are distinct from ADCs in that ON linker-payloads are larger than typical drug-linker moieties and are negatively charged. Once conjugated, ADCs typically have the same or similar quality attributes as the mAb intermediate, with the exception that the hydrophobicity of the drug-linker may result in higher levels of aggregates in the ADC compared to the mAb. On the other hand, AOCs display properties of both the mAb and ON intermediates. Conjugation chemistry will be different and there will be changes in the charge profile of the mAb (96–98). Therefore, the typical reversed-phase HPLC and hydrophobic interaction chromatography methods used to measure the drug to antibody ratio cannot be used to determine the oligo to antibody ratio (OAR) (96).

In addition to new types of payloads, much effort is going into improving linker technologies, which fall into two broad categories, cleavable and non-cleavable. Typically, an ADC is internalized into cells where the payload is released to kill the cell via the mechanism of the payload. How and when the payload is released is dependent on the linker design. There are many excellent reviews that cover linker development (99–102). Ideally the linker will be stable in circulation to minimize toxicity and maximize payload delivery to the tumor. The two approved ADCs with topoisomerase 1 inhibitor payloads, sacituzumab govitecan and fam-trastuzumab-deruxetan, provide interesting examples.

Sacituzumab govitecan, an anti-Trop 2- CL2A-SN38 ADC with an average DAR of 7-8 uses a hydrolysable linker where the SN38 payload is released in serum at 37°C with a half-life of ~1 day. This release rate was determined to be important for activity and results in a bystander effect on nearby cells in addition to killing by internalization (103).

Fam-trastuzumab-deruxetan, with an average DAR of 8, is approved for tumors with variable levels of HER2 expression, unlike other HER2 targeted mAbs. The linker is stable and upon internalization, is cleaved by lysosomal enzymes. Due to the DAR of 8 which delivers more drug, and membrane permeability of the deruxetan moiety, it can kill nearby tumor cells regardless of HER2 expression (104).

Like BsAbs, newer designs of ADCs address on-target/off tumor toxicity using prodrug formats. The binding sites of the mAb are masked with a peptide linked to a protease substrate that is cleaved in the TME, while the payload is unprotected (105). These ADC prodrugs are designed to widen the therapeutic index of ADCs and possibly broadening the mAb targets. One product targets CD71 (transferrin receptor), which is highly expressed on tumors, but also expressed widely on normal tissues. It showed growth inhibition of different tumor types in xenograft mouse models and an acceptable tolerability and safety profile in cynomolgus monkeys (106). It also showed clinical activity and acceptable tolerability in a Phase 1 clinical trial (107).

### 3.3 Mab conjugates for imaging and therapy

Many of the earliest clinical studies of mAbs used radiolabeled murine mAbs for imaging or as radioimmunotherapy (RIT). Five of the 6 mAbs approved between 1991 - 1996 were imaging agents labeled with 111-indium or 99-technetium. However, these were not commercially successful, likely due to being murine mAbs as well as other advances in imaging technologies.

Two murine anti-CD20 RITs, 90-yttrium ibritumomab tiuxetan and 131-iodine tositumomab, were approved in 2002 and 2003, respectively. Only 90-yttrium ibritumomab tiuxetan remains on the market, but it is not widely used (108). The dosing regimen for both products included pretreatment with rituximab (for 90-yttrium ibritumomab tiuxetan) or tositumomab (for 131-iodine tositumomab) prior to administration of a dosimetric dose (111-Indium Zevalin or a lower dose of ^131^I- tositumomab) to improve targeting to the tumor and to assess biodistribution. After several images were collected, the therapeutic dose of either product was administered about a week later, also preceded by pretreatment with rituximab or tositumomab. Based on supporting data, the ^111^In- ibritumomab tiuxetan imaging dose was removed from the label in 2011. In general, RITs are more complicated to administer to patients as they require a reliable source of clinical and GMP grade radionuclide, referral to a nuclear medicine/radiation oncologist and manufacturing on site for radionuclides with a short half-life, such as ^90^Y- ibritumomab tiuxetan.

Although imaging agents and RITs have not been as successful as unlabeled mAbs, continued development of these products parallels the progress for ADCs. This includes new “payloads” such as alpha emitters (225-actinium), PET imaging agents (64-copper, 89-zirconium), and optical imaging probes (CY5.5, IRDyes), linker/chelator and conjugation technology, determining the appropriate label-linker/chelator combination and whether to use an intact mAb or mAb fragment (109–112). IRDye-mAb conjugates are also used as photoimmunotherapy, where after injection of the mAb conjugate and localization to the tumor, the dye is activated using an external laser, killing targeted cells. Cetuximab saratolacan was approved in Japan in 2020 (113). CQAs for radiolabeled mAbs and optical imaging probes will depend on the conjugation/chelation strategy, but may include the immunoreactivity before and after labeling, sites and number of sites of conjugation/chelation per mAb, free mAb and free radionuclide or label. In addition, since radiolabeled drug products may be administered to patients prior to final quality control testing of the product, for an initial IND submission, it is recommended that data be provided from two to three radiolabeling runs that demonstrate the preparation of an immunoreactive, sterile, and pyrogen-free product.

Challenges for the development of imaging agents and RITs are like other mAb based products, especially for solid tumors (114–116). Optimizing the format and affinity depends on the intended use. MAb fragments that penetrate tumors better than full length mAbs may be preferred for imaging agents, while full length mAbs with longer half-lives may be better in some cases for RIT and for optical imaging agents that emit their signal upon binding the tumor target (109, 111, 112). Optimizing the affinity will depend on the choice of mAb design, linker/chelator, and half-life of the radionuclide, and will be unique for different products. For optical imaging, the charge and other properties of the dye and choice of linker may impact the PK of the conjugate, the tumor to background ratio and aggregation of the mAb (117–119).

The development of imaging agents is largely by academic rather than commercial sponsors. It remains to be seen if there will be a commercial market for these products, but one use is for guided surgery to better identify cancer tissue for resection (120, 121). RITs are in development by both academic and commercial sponsors. An anti-CD45 mAb,^131^I-apamistamab, is in Phase 3 clinical trials for acute myeloid leukemia (122) and an anti-PSMA mAb,^177^Lu-rosopatamab, is in Phase 3 clinical studies for metastatic castrate-resistant prostate cancer (123). However, the direction of radiotherapy seems to be towards smaller molecules such as peptides and small molecule drugs, as seen with the approvals of Lutetium Lu 177 Dotatate and 177Lu-PSMA-617 (124, 125). For mAb-based products, future development in RIT may favor mAb fragments, but full length mAbs may still be a better option for some indications.

### 3.4 MAb fragments, fusion proteins, and other constructs

As noted above, mAb fragments can be used as building blocks for BsAbs and conjugates but are also stand-alone monospecific products, either as monomers or engineered as multimers. Although several approved mAbs are fragments (Fab, single domain, sFv), the only approved mAb-fragments for oncology are scFv-based BsAbs (blinatumomab and tebentafusp). The half-life of fragments may be too short to provide efficacy when targeting cell surface receptors; however, single domain antibodies targeting ligands such as VEGF, HGF or EGF showed promise in non-clinical studies both as imaging agents and therapeutics (126–128). *In vitro* studies also showed that anti-EGF single domain antibodies enhanced the activity of osimeritinib, an EGFR tyrosine kinase inhibitor (129).

Intact mAbs and fragments are also in development as fusion proteins. These are bifunctional molecules that have the binding activity and possible Fc-effector function of a mAb combined with the activity of the protein fused to the mAb. Among mAb-fusion proteins, immunocytokines are a growing class of products. Cytokines have promising efficacy in some indications, but unacceptable toxicities (130, 131). Fusion with mAbs allows targeting cytokines such as IL-2 or IL-12 to the tumor to broaden their therapeutic index. The cytokine, such as IL-2, may have other modifications to favor binding to one type of IL-2 receptor (132) or may be in prodrug form where fusion protein consists of a cytokine and the binding portion of the receptor, which blocks activity of the cytokine. The cytokine becomes active in the TME upon cleavage by matrix metalloproteinases (MMP) (131, 133). IL-15 mAb constructs include the minimal binding domain of the IL-15Ra chain, which enhances interaction with the common IL-2Rβ and IL-2Rγ chains to stimulate NK and memory CD8+ T cells (134). The mAb portion of the immunocytokine typically targets a tumor antigen and may be used in combination with a checkpoint inhibitor mAb, but anti-PD-1 mAbs have been used in the immunocytokine constructs (130).

Non-immunoglobulin scaffolds are small single domain units derived from a variety of proteins, where the structure is amenable to engineering loops between strands of the structure to mimic the CDR regions of antibodies (135, 136). Like single domain abs, these structures are used as building blocks for monospecific or multispecific larger structures (137). Like single domain abs and fragment based BsAbs, they often include an anti-HSA domain, HSA itself or are fused to an Fc region to extend half-life.

There are some common quality attributes for the variety of structures described in this section. Whether they are CQAs should be determined during clinical development of the product. The small mAb fragments and non-Ig scaffolds are typically non-glycosylated and are easier to characterize than full length-mAbs. However along with mAb-fusion proteins, these products often link the different domains via Gly-Ser (GS) linkers, which can be subject to O-glycosylation and other post-translational modifications (138–141). Post-translational modifications of the linkers should be part of the characterization of these products.

In addition to the potential O-glycosylation of the GS linker in mAb-fusion proteins, the fusion protein often has more complex N- and O-glycans compared to the IgG Fc-glycan structure. The glycans on both the mAb and the fusion protein should be characterized separately and included in the control strategy, if necessary.

For immunocytokines, binding to the target and the activity of the cytokine are CQAs and should be incorporated into the control strategy. If the mAb has Fc-mediated effector functions, this CQA should also be included in the control strategy. For constructs where the cytokine is in a prodrug configuration, the cytokine activity will be minimal. Therefore, the bioassay controlling cytokine activity should be performed on both the prodrug form and the product after treatment with an MMP. This concept also applies to prodrug BsAbs and ADCs for demonstrating binding to the target after treatment with the appropriate protease.

## 4 Immunogenicity

Antibodies are inherently immunogenic as suggested by the anti-idiotype network theory (142). The immunogenicity of mAbs is due to both patient and drug related causes (143). Patient-related causes include the nature of the disease, genetic background and concomitant medications. Drug-related causes include the mAb origin and engineering design, target antigen, post-translational modifications, impurities, formulation, dose, route of administration, and frequency of dosing. MAbs of rodent or other non-human origin are generally the most immunogenic, followed by chimeric, humanized and human mAbs.

Immunogenicity cannot be directly compared across products because the ADA assays are specific for each mAb. In addition, today’s assays are more sensitive compared with the ADA assays used 20 years ago and can better detect ADA than older methods. However, given the prediction that the more human a mAb, the less immunogenic, an analysis of ADA responses by origin suggests that human and humanized mAbs may not be significantly less immunogenic overall when compared with chimeric mAbs. This is likely due to patient factors as well as the specific amino acid sequences and engineering of mAbs. Some murine mAbs have low rates of ADA, which may be due to the target and dosing regimen.

Based on information provided in product labels, the formation of anti-drug antibodies (ADA) in oncology indications generally appears to be low. This may be due to concomitant immunosuppressive medications and use in late-stage cancer indications. However, higher rates of ADA can be seen for some products.

**Figure 3:**
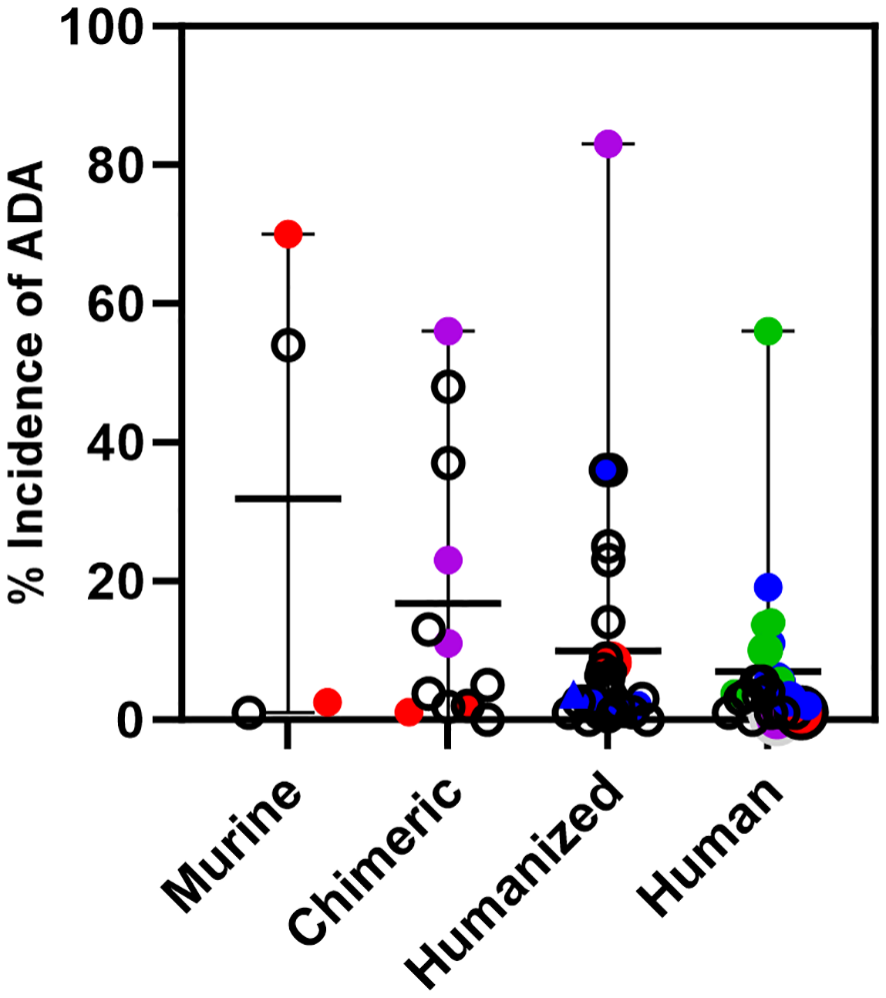
Immunogenicity of monoclonal antibodies approved for oncology indications. Incidence of ADA is shown for oncology mAbs of murine, chimeric, humanized, or human origin and includes the incidence for those mAbs that are also approved for non-oncology indications. Murine mAbs: Incidence of ADA for ibritumomab and tositumomab, red circles. Chimeric mAbs: Incidence of ADA for rituximab and rituximab hyaluronidase in oncology, red circles, and for rituximab in autoimmune indications, purple circles. Humanized mAbs: Incidence of ADA for alemtuzumab in oncology, red circle and an autoimmune indication, purple circle. Incidence of ADA of CKI mAbs; pembrolizumab, atezolizumab, dostarlimab, retifanlimab, and toripalimab, blue circles. Human mAbs: Incidence of ADA for ofatumumab in oncology, red circle and an autoimmune indication, purple circle. Incidence of ADA of CKI mAbs when used as monotherapy; ipilimumab, nivolumab, durvalumab, avelumab, cemiplimab, blue circles. Incidence of ADA of CKI mAbs when used in combination; ipilimumab, nivolumab and durvalumab, green circles. The incidence of ADA for a CKI mAb can vary per indication. For those with multiple indications, only the highest incidence of ADA seen with monotherapy or in combination is shown.

Using data taken from the labels of approved therapeutic oncology mAbs (see **Supplementary Table 1**), ADA for murine (4), chimeric (9), humanized (24), and human mAbs (18) highlight several points (**Figure 3**). For murine antibodies, an example of product factors may be seen with two anti-CD20 radioimmunoconjugates, ^90^Y-ibritumomab tiuxetan and ^125^I-tositumomab, which had 3.8% and 70% ADA, respectively. The dosing regimen for both includes pre-dosing with cold antibody to ensure the radiolabeled product targets the tumor rather than normal B cells. Patients treated with ^90^Y-ibritumomab were pretreated with rituximab, the chimeric version of ibritumomab, while patients treated with ^125^I-tositumomab were pretreated with unlabeled tositumomab. The lower levels of ADA seen with ibritumomab may be due to pretreatment with a chimeric mAb rather than a murine mAb.

An example of patient factors is seen with chimeric and humanized antibodies approved for both oncology and autoimmune diseases. Oncology patients treated with rituximab, and rituximab co-formulated with recombinant hyaluronidase have ADA levels ≤2.0%, while in the autoimmune indications, patients treated with rituximab have ADA ranging from 11-56%. Similarly, alemtuzumab, a humanized mAb, has low rates of ADA in the oncology indication (2%), but high rates in patients with multiple sclerosis (83%). On the other hand, the human mAb ofatumumab, which is also approved for oncology and multiple sclerosis has low levels of ADA in both patient populations. It is not clear if the human origin of ofatumumab is the only reason for low levels of ADA in the multiple sclerosis indication, but levels of ADA detected for other human mAbs approved only for autoimmune indications range from <1% up to 61% (data not shown). The variability in rates of ADA in autoimmune indications for human mAbs likely reflects both product and patient factors.

Several approved humanized and human mAbs target immune checkpoints (CKI) such as PD-1 (6 mAbs), PD-L1 (3 mAbs), CTLA-4 (2 mAbs) and LAG-3 (1 mAb). These mAbs are approved for many different cancer indications as monotherapy and some are approved in combination with other CKI mAbs (nivolumab plus ipilimumab, nivolumab plus relatlimab and durvalumab plus tremelimumab). Relatlimab and tremelimumab are only approved for combination use. The rates of ADA for any given CKI mAb vary per indication and treatment regimen.

In **Figure 3**, only the highest rate of ADA for a given CKI across indications is shown. For the humanized mAbs the highest incidence of ADA across indications for each mAb was <4.0% for pembrolizumab, dostarlimab, retifanlimab, and toripalimab, and 36% for atezolizumab. For the human mAbs given as a single agent, the highest incidence of ADA across indications was 5.4% for ipilimumab, 11% for nivolumab, 3% for durvalumab, 19.1% for avelumab, 2% for cemiplimab.

However, for CKI mAbs used in combination, the rates are higher compared to their use as a monotherapy, For the combination of nivolumab and ipilimumab, the highest incidence of ADA for nivolumab was 56% in hepatocellular carcinoma and 13.7% for ipilimumab in malignant pleural mesothelioma. For the combination of durvalumab and tremelimumab, the highest incidence of ADA for durvalumab was 10% and 14% for tremelimumab in metastatic non-small cell lung cancer. These data show that in oncology indications where the products activate the immune system to target the cancer, an activated immune system can generate ADA against the therapeutic mAb, even of human origin.

Fc-engineered mAbs and mAb-based products that are not basic Ab structures are not found in nature and may provoke an ADA response against the engineered amino acids or different subunits of the construct, whether it be a BsAb, conjugate or fusion protein. The assays should be designed to detect ADA against each component of the product. There may be pre-existing ADA against elements of some constructs that are based on a platform. These complex molecules should be tested against normal human serum to determine if there are pre-existing ADA against any component of the product. For example, there may be pre-existing anti-hinge region antibodies, especially in rheumatoid arthritis patients, that may bind to mAbs and Fc-fusion constructs (144) or against the CH3 domain of IgG4 (145).

Finally, the impact of pre-existing and emergent ADA targeting different domains of mAbs, complex mAb-based products or engineered Fc region mutations on the safety and efficacy of a product should be considered when planning clinical trials. Depending on the mAb and indication, the ADA may affect PK, may be neutralizing or may have no effect. For most oncology mAbs, there has been no identified clinically significant effect of binding or neutralizing ADA on PK or safety but the effect on efficacy in any given patient is unknown.

## 5 Future development

Next generation IgG mAbs, fragment-based mAbs, and mAb-based multi-specific conjugate and fusion proteins will build on the lessons learned from both failed and successful products. Some will be more complex constructs such as bispecific ADCs, bispecific fusion proteins, or ADCs with linkers containing many drugs or more than one type of drug. The next generation mAbs will have new challenges for manufacturing, characterization, understanding the CQAs and development of an appropriate control strategy.

In addition to these products, other isotypes, such as IgM, IgE, IgA and IgG3 have been in preclinical and clinical development over the years, including for oncology indications (146–153). The CQAs for these isotypes are not as well understood as those for IgG1, IgG2 and IgG4 mAbs, leading to challenges for both the manufacture and development of a control strategy for these isotypes.

Among the IgG sub-isotypes, IgG3 has the best binding to FcγRs and the highest CDC activity but has 29 allotypes, including different lengths of the hinge region, and most allotypes have a short half-life of ~7 days. It is O-glycosylated and depending on the allotype, can have up to 11 inter-chain disulfide bonds between H chains (154, 155). These features make IgG3 mAbs more challenging to design, characterize and manufacture with consistency. The short-half life can be circumvented by using one of the natural allotypes with a histidine at position 435 in CH3 that has a half-life comparable to IgG1 (156). This substitution also improves binding to Protein A and in combination with two other mutations in CH3 that reduce aggregation, the manufacturability of IgG3 mAbs can be improved (157).

IgM (146), IgA (158) and IgE (159) all have more complex structures than IgG including: 4 constant region domains for IgM and IgE; multiple N-glycosylation sites on all 3 isotypes; O-glycosylation on IgA and IgE; pentameric and hexameric IgM; dimeric IgA; J chain complexed with pentameric IgM and dimeric IgA: and secretory component derived from the polymeric Ig receptor in addition to the J chain complexed with secretory IgM and IgA. All three isotypes have their own sets of Fc receptors (152, 158, 160, 161), which are expressed or may be induced on different effector cells, as well as cell types other than those that express FcγR (See Table 1 in Liu et al. (162)).

As these isotypes progress through clinical development, a thorough characterization of the mAbs, including a determination of potential Fc-mediated effector functions and effector cells engaged by these isotypes will be needed to understand those quality attributes that should be included in the control system and how best to engineer the isotype for a specific purpose in the way that is now possible for IgG mAbs.

The success of mAbs comprises many bench-to-bedside, back to the bench and back to the beside stories. Lessons learned from each generation of product are applied to the next generation, leading to more approved products. But challenges and failures with each generation of product remain. If each new generation of mAbs successfully incorporates designs that overcome previous challenges, more patients will benefit from mAbs.

## Author contributions

MS: Writing – review & editing, Writing – original draft.
